# Active Navigation in Virtual Environments Benefits Spatial Memory in Older Adults

**DOI:** 10.3390/brainsci9030047

**Published:** 2019-02-26

**Authors:** Melissa E. Meade, John G. Meade, Hélène Sauzeon, Myra A. Fernandes

**Affiliations:** 1 Department of Psychology, University of Waterloo, Waterloo, ON N2L 3G1, Canada; jon.meadee@gmail.com (J.G.M.); mafernan@uwaterloo.ca (M.A.F.); 2 Lab EA413-Handicap, Activité, Cognition & Santé, Université de Bordeaux, F-33000 Bordeaux, France; helene.sauzeon@u-bordeaux.fr; 3 Flowers Team-INRIA Center, F-33405 Talence, France

**Keywords:** aging, spatial memory, active exploration, virtual reality

## Abstract

We investigated age differences in memory for spatial routes that were either actively or passively encoded. A series of virtual environments were created and presented to 20 younger (Mean age = 19.71) and 20 older (Mean age = 74.55) adults, through a cardboard viewer. During encoding, participants explored routes presented within city, park, and mall virtual environments, and were later asked to re-trace their travelled routes. Critically, participants encoded half the virtual environments by passively viewing a guided tour along a pre-selected route, and half through active exploration with volitional control of their movements by using a button press on the viewer. During retrieval, participants were placed in the same starting location and asked to retrace the previously traveled route. We calculated the percentage overlap in the paths travelled at encoding and retrieval, as an indicator of spatial memory accuracy, and examined various measures indexing individual differences in their cognitive approach and visuo-spatial processing abilities. Results showed that active navigation, compared to passive viewing during encoding, resulted in a higher accuracy in spatial memory, with the magnitude of this memory enhancement being significantly larger in older than in younger adults. Regression analyses showed that age and score on the Hooper Visual Organizational test predicted spatial memory accuracy, following the passive and active encoding of routes. The model predicting accuracy following active encoding additionally included the distance of stops from an intersection as a significant predictor, illuminating a cognitive approach that specifically contributes to memory benefits in following active navigation. Results suggest that age-related deficits in spatial memory can be reduced by active encoding.

## 1. Active Navigation in Virtual Environments Benefits Older Adults’ Spatial Memory

Spatial navigation deficits are frequently experienced in the older adult population [1,2,3]. For example, age-related deficits are commonly observed in the memory for locations, landmarks, routes, and maps [2,4]. Such declines in navigation and spatial memory are critical, because they pose a potential threat to independent living for older adults [5,6,7,8,9], prompting the need to find ways to lessen these age-related deficits. The assessment of age-related impairments in spatial cognition has been facilitated by recent advancements in virtual reality (VR) technology [10], allowing for the efficient examination of navigation performance in virtual environments. In the current study, we took advantage of VR methodology to investigate ways for improving spatial memory performance in a navigation task, in older and younger adults. Specifically, the goal of the current study was to determine whether age-related differences in memory for virtual routes are reduced following active exploration, compared to passive guidance. Additionally, we examined whether individual differences in visuo-spatial ability, and cognitive indices during route exploration, meaningfully contributed to spatial memory performance. 

## 2. Active vs. Passive Encoding of Spatial Information

The term active navigation refers to a condition wherein the participant has motor and/or volitional control of their movement through an environment, whereas passive navigation generally consists of a guided tour [11]. Active navigation has been described to contain both cognitive (mental manipulation of spatial information, allocation of attention, and decision-making) and physical (motor control for locomotion and proprioceptive and vestibular sensory information) components [11], which combine to create rich multi-modal associations that benefit subsequent memory performance [12]. A variety of past studies in young adults suggest significant spatial memorial benefits when encoding is active, rather than passive [13,14,15,16]. 

The memory benefit from active, relative to passive, navigation can be conceptualized as a subject-performed task effect (SPT effect) [17,18]. The SPT effect describes a pattern of superior memory performance when encoding involves the direct engagement of the participant, such as when performing rather than watching an action that is associated with a to-be-remembered word [19]. Importantly, the SPT effect remains unchanged with aging, enhancing memory performance in older adults [20,21,22]. The encoding tasks that involve an SPT have been described to have a high degree of environmental support, accounting for a boost in memory in seniors [23,24]. With regard to encoding manipulations, the environmental support refers to a situation in which older adults do not need to self-initiate encoding operations. By this account, active navigation should provide a good form of environmental support, as it involves the SPT of physically and cognitively engaging with the to-be-remembered spatial information. Past studies, however, have reported inconsistent results regarding the benefits of active relative to passive navigation in older adults. Specifically, benefits from active navigation are sometimes observed in older adults [15,25,26], but there is also evidence showing that active encoding hinders memory performance in seniors [2,27]. 

The degree of involvement of both decision-making processes and motor processing in active exploration conditions could be critical for explaining the inconsistencies regarding age differences in active/passive manipulation. It is well-known that older adults have to allocate additional cognitive resources to control their locomotion [28,29], suggesting that the physical components of active navigation are unlikely to be the reason for any memory benefit in seniors. Indeed, active navigation with low levels of motor control benefits spatial memory, whereas a high degree of motoric requirement creates an additional cognitive load that impairs memory in older adults [27]. 

Some have suggested that the cognitive components of active navigation benefit the memory (for directions and the locations of landmarks), more so than physical motor processing [29,30,31]. Decision-making in particular has been shown to be beneficial in older adults for episodic memory of landmarks and objects [15] and it may elicit a self-reference effect that is known to benefit older adults’ spatial memory [32,33,34]. In other words, the engagement of cognitive and decision-making processes during active navigation conditions could be particularly beneficial for older adults. Likewise, factors related to decision-making, such as intrinsic motivation or curiosity [35,36] may also contribute to the memory benefit from active exploration. As recently claimed by [37], momentary feelings of curiosity can help older adults’ memories and executive functions, because the phasic activation of the noradrenergic and dopaminergic systems modulate hippocampal activity [38], facilitating memory and learning. As such, it may be that the cognitive components of active navigation drive the observed active encoding memory benefits in older adults.

Related to this, it is possible that the cognitive processes engaged by the active exploration of an environment allow for more unique associations to be formed with landmarks, and thus enhance the memory for the links between certain environments and paths travelled. Others have suggested that such relational knowledge is critical, as retrieval becomes more likely when the target is part of a rich network of associations, as opposed to being encoding in isolation [12]. This relational encoding can take the form of distinctive sensorimotor associations, and increased elaboration during encoding, due to goal-directed exploration. By actively encoding an experience, and thereby increasing the amount of associations that are credited to an episodic memory, later memory recall can be expected to be improved [12].

## 3. Using Virtual Reality to Examine Spatial Memory

In recent years, VR technology has become widely used for studying spatial cognition and navigation performance in both younger [39,40,41,42], and older adults [10,12,43]. One of the major advantages of VR is the high degree of experimental control that is afforded, to investigate the cognitive and behavioral components that are involved in spatial navigation. For example, VR provides the ability to control for variables that differ between age groups, such as physical fatigue and mobility issues during navigation. Furthermore, the use of VR in the current study allowed for the creation of a series of environments with controlled parameters, including environment size, number of intersections, and visual content, as well as the novel approach of examining spatial memory following trials of either active or passive encoding. Additionally, we developed a means to precisely measure the overlaps in the routes travelled at encoding and retrieval, to provide a measure of memory accuracy in re-tracing a previously travelled route.

Research into the effects of aging on route navigation have largely focused on differences in learning performance and the assessment of knowledge for a highly familiar environment. However, it is not the case that we will only ever need to navigate through familiar environments; rather we frequently encounter new spaces by we need to successfully navigate after brief encounters. As such, in the present study, we took the novel approach of investigating the age difference in memory for routes that were previously travelled on only one occasion. We created a set of virtual environments in which participants traveled routes, and then later re-traced those routes to assess spatial memory performance. During the encoding, participants either traveled routes by actively exploring through volitional control of their own movements, by passively viewing a guided tour. This paradigm was analogous to drivers and passengers in a car, with active trials affording the control that a driver has in deciding on the route and manipulating movement, whereas the passive trials are akin to a passenger simply viewing the route travelled within the environment. We expected that our active encoding manipulation would provide a higher degree of environmental support, as it constitutes a SPT; given that environmental support is particularly helpful to seniors [23,24], active versus passive encoding should benefit older adults’ memories more so than younger adults. 

## 4. Method

### 4.1. Participants 

Twenty-two younger (M_age_ = 19.71, SD = 2.19, 11 females) and 22 older (M_age_ = 74.55, SD = 7.82, 10 females) adults participated in our study. Younger adults were undergraduate students at the University of Waterloo who signed up for the study and received partial course credit for their participation. Older adults were recruited from the Waterloo Research in Aging Participant (WRAP) database, and received $10.00 remuneration for their participation. WRAP is a database of healthy seniors residing in the Kitchener–Waterloo area recruited by means of newspaper ads, flyers, and local television segments to participate in research studies taking place on campus at the University of Waterloo. All older adults completed the Montreal Cognitive Assessment (MoCA) to assess cognitive decline [44]. The average MoCA score was 26.95 out of 30 (SD = 2.29), with a range of 20 to 30. Two participants fell below the cut-off a score of 26 (obtaining scores of 24 and 20); which was generally used to indicate normal healthy aging; however, we opted to include all older adults who were tested, as we are not aiming to make a distinction between normal healthy aging and MCI in the current study. 

### 4.2. Materials

**Figure 1:**
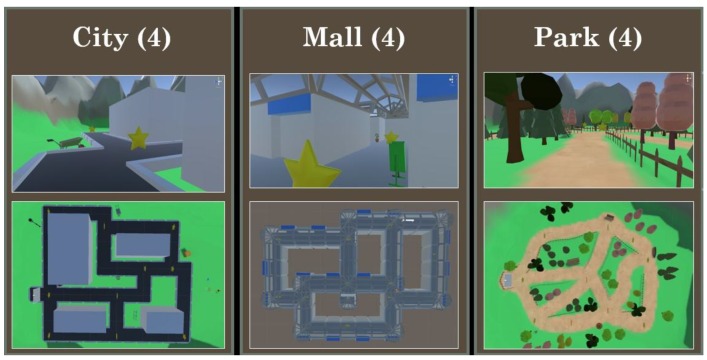
First-person view and bird’s-eye view of the three virtual environment styles. For each style, we created 4 exemplars, though only one from each is shown here. The Stars are visible in the environments during encoding, but not during retrieval. Participants only experienced the environments from a first-person perspective.

VR equipment. The VR program was run on an Asus© Zenfone 3 Laser ™ (see specificities on www.asus.com, Asus, Taipei, Taiwan) and a lightweight hand-held cardboard VR headset for viewing. The VR application run on the Android phone was designed using the 3D engine Unity© (ver. 2017.2.0.f3, Unity Technologies ApS, San Francisco, CA, USA). Twelve virtual environments (VE) were built according to three styles of: city streets, mall hallways, and park trails (see Figure 1 for samples). The virtual environments were topographically similar, with an average area of 234 square meters. Additionally, each environment had six intersections, which is a point where 3+ roads converge. 

Neuropsychological assessments. Spatial visualization ability was evaluated with the Hooper Visual Orientation test [45], which had good internal reliability, as indicated by a coefficient α of 0.88 [46]. This test is scored out of 30; it contains 30 line drawings of common objects that are portrayed as having been cut up and misaligned; participants had to mentally rotate and piece together the visual information, to identify the depicted object. Performance on the Hooper test was included to determine whether spatial visualization abilities were related to, and predictive of, spatial memory performance following active and passive encoding. 

The spatial orientation ability was measured with the Santa Barbara Sense-of-Direction scale, which has good internal reliability, as indicated by a coefficient α of 0.88 [47]. For this, the test participants rated their endorsement of 15 statements about spatial orientation abilities, such as “I am good at giving directions’, indicating whether they strongly agreed or disagreed, using a 7-point scale. After reverse scoring the necessary items, the responses were summed and divided by 15 to provide an average score of between 1–7 [47]. This measure was included to determine whether the perceived spatial orientation abilities were related to spatial memory performance following active and passive encoding.

## 5. Procedure 

The study received clearance from the ethics review board at the University of Waterloo; all participants gave their written informed consent prior to beginning the experiment. Throughout the entire experiment, participants were seated in comfortable chairs that could turn a full 360° circle, which was placed in the center of a testing room. Following a verbal explanation of how to use the VR headset, participants were given unlimited time to complete a training phase with the VR equipment designed to help them learn how to move in the virtual space. Specifically, they were told that to move forward in VR, they should press a button on the headset, and to change their direction within VR, they were to physically rotate in the spinning chair with their legs. Participants were advised to take as much time as needed in the training phase, to become comfortable with the environment and the movement mechanism. Immediately following the training phase, the experimental session began. 

In the experimental session, participants completed 12 cycles of encoding-filler-retrieval phases. The encoding phase was 30 seconds in duration. Once 30 seconds elapsed, the screen turned black and the participant immediately performed a filler task which involved counting backwards by 7’s from 100, for 20 seconds, in order to reduce recency effects [48]. In the retrieval phase, participants were placed within the VR environment at the same starting point as the environment they had just experienced. Participants were told that they had 30 seconds to re-trace the exact path just previously travelled in the environment. Participants were given a 1- to 5-min break halfway through the experimental session (i.e., after completing six encoding-filler-retrieval cycles), resuming when they indicated they were ready.

Active vs. passive manipulation. The within-subjects active/passive navigation manipulation was implemented during the encoding phase. Of the 12 virtual environments, six were actively encoded, and six were passively encoded. The order of active and passive trials was mixed, with an instruction appearing at the beginning of each new encoding-filler-retrieval cycle, indicating whether the participant was to explore the environment (active) or watch a guided tour (passive). Participants knew that they would subsequently be asked to retrace the travelled route. In the active trials, the participant had full volitional control of their movement along the paths in the environment, and they were free to choose the route they traveled. To motivate participants to continue to move and explore the environment in the active condition (and not to simply walk back and forth so that their route was later easier to remember) they were told to ‘collect’ as many star icons along the path as possible, by walking over the stars. Each environment had 10 stars dispersed throughout the environment, along the paths (as shown in Figure 1). On the passive trials, the participant did not control their movement, or route, in the environment, and instead experienced a recording from the active exploration of a previous participant. Using a previous participant’s active exploration for the passive trials, in this yoked control manner, was purposely done to equate route length, pauses along the path, and the number of turns, across the active and passive trials. 

## 6. Results

Participant Characteristics. While age differences in spatial abilities were of primary interest in the current study, we also wanted to determine whether any sex differences that we presented in our data, given the previous work, suggested that males perform better than females on a variety of spatial processing measures [49]. To determine whether there were differences between the age groups and sexes, on the neuropsychological assessments included in the study, we ran two 2 (Age: younger, older) × 2 (Sex: male, female) ANOVAS separately, for the performance on the Hooper Visual Orientation test [45] and on the Santa Barbara Sense-of-Direction scale [47]. The analysis on the Hooper Visual Organization performance revealed a main effect of Age, *F*(1, 40) = 26.12, *MSE* = 0.26, *p* < 0.001, and *η*^2^ = 0.40, such that the scores were higher for younger than for older adults, but there was no effect of Sex, *F*(1, 40) = 2.09, *MSE* = 0.02, *p* = 0.16, η^2^ = 0.05. There were no main effects of Age, *F*(1, 40) = 0.06, *MSE* = 0.14, *p* = 0.80, *η*^2^ = 0.002, or Sex, *F*(1, 40) = 0.48, *MSE* = 1.07, *p* = 0.50, *η*^2^ = 0.01 on the Santa Barbara Sense-of-Direction scale, and no interactions on either measure.

**Figure 2:**
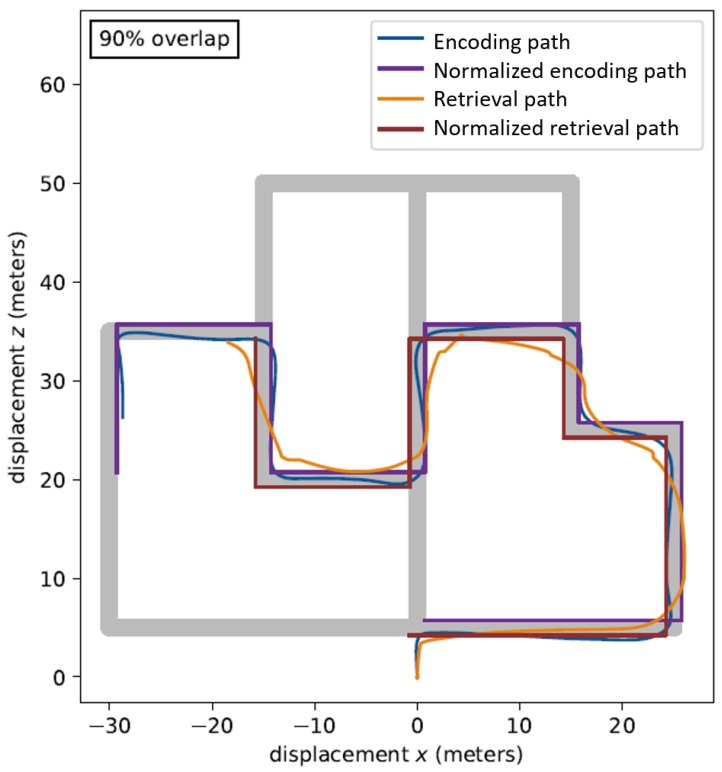
Example of the paths travelled in one of the virtual environments at encoding and retrieval before and after normalization.

Calculation of Route Overlap. The dependent variable of interest was the accuracy in re-tracing the same route at retrieval that had been traveled during encoding. To obtain this measure of spatial memory performance, the overlap between the routes travelled by each participant at encoding and retrieval, for each trial type, was calculated, to produce a ‘percent overlap’ value for each environment. The percent overlap values were computed by using the Python programming language (https://www.python.org/), Python Software Foundation, Wilmington, DE, USA), to first normalize the routes travelled (during both encoding and retrieval) against the established paths in the environments, and then to compare those normalized routes. The process of normalizing the participant’s paths involved removing positional data points that corresponded to the subject travelling between specified points on the pathways in the environments. The normalized path that this process produced is a distilled representation of the position data, which allowed us to measure the overlap values that were not obscured by other effects (such as a participant travelling in a straight line during encoding, versus a sinusoidal path in retrieval). See Figure 2 for a visual representation of the normalized paths used in the calculation of the Route Overlap values. This process resulted in 12 values for percent overlap scores per person, 6 of which corresponded to active trials, and six to passive trials. For each participant, the averages were calculated for the active and passive trials separately, resulting in one measure representing performance following active encoding, and another following passive encoding. See the example in Figure 2.

Route overlap analyses. A mixed measures ANOVA (Analysis of variance) (2 (Age: younger, older) × 2 (Trial type: active, passive) × 2 (Sex: male, female)) was conducted on the Route Overlap values, with the Trial type as a within-subjects factor, and Age and Sex as between-subjects factors. The analysis revealed a significant main effect of Trial type, *F*(1, 40) = 23.07, *MSE* = 0.27, *p* < 0.001, *η*^2^ = 0.37, such that the Route Overlap was more accurate for active than passive encoding. A main effect was also found for Age, *F*(1, 40) = 83.44, *MSE* = 1.99, *p* < 0.001, *η*^2^ = 0.68, such that the Route Overlap was higher for younger than for older adults. No main effect was found for Sex, *F*(1, 40) = 0.03, *MSE* = 0.001, *p* = 0.86, *η*^2^ = 0.06. There was a significant two-way interaction between Trial type and Age *F*(1, 40) = 5.65, *MSE* = 0.07, *p* = 0.03, *η*^2^ = 0.12. The Age × Sex interactions was non-significant, *F*(1, 40) = 2.42, *MSE* = 0.06, *p* = 0.13, *η*^2^ = 0.06, as was the Trial type × Sex, *F*(1, 40) = 1.38, *MSE* = 0.02, *p* = 0.25, *η*^2^ = 0.03. The 3-way interaction was also non-significant, *F*(1, 40) = 0.38, *MSE* = 0.005, *p* = 0.54, *η*^2^ = 0.01. 

**Figure 3:**
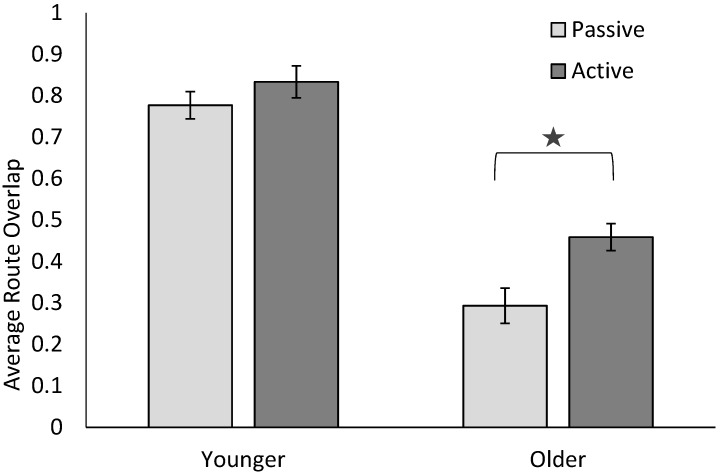
Average percent Route Overlap values for active and passive encoding in younger and older adults. Error bars represent the standard error. The star represents significant differences between active and passive conditions for older adults.

To better understand the interaction between Age and Trial type, two paired-sample *t*-tests were conducted on with Route Overlap accuracy, separately for younger and older adults. For younger adults, there was a marginally significant difference, *t*(21) = 1.98, *p* = 0.06, *d* = 0.35, such that the Route Overlap was higher following active encoding, compared to passive encoding. For older adults, the Route Overlap was significantly higher following active encoding, compared to passive encoding, *t*(21) = 4.55, *p* < 0.001, *d* = 0.61. See Figure 3 for the mean values.

Correlations with neuropsychological assessments. To determine whether spatial memory performance was related to performance in the Hooper Visual Orientation test [45] and the Santa Barbara Sense-of-Direction scale [47], we ran a series of partial correlations separately, for active and passive encoding, while controlling for age. A significant and positive relationship was found between Route Overlap accuracy and the Hooper Visual Orientation test, for both active (*r* = 0.51, *p* = 0.001) and passive (*r* = 0.42, *p* = 0.005) Trial types, suggesting, as expected, that better spatial visualization abilities are related to superior spatial memory performance, regardless of whether one actively or passively encodes a route. Correlations with the Santa Barbara Sense-of-Direction scale were non-significant.

Correlations with cognitive indices. Given our goal of determining the factors underlying the benefits of Active encoding, we conducted exploratory analyses on a number of measures obtained from our virtual reality program. Specifically, a number of partial correlations were analyzed. Table 1 shows the factors considered, along with the correlation and the statistical significance values. Given the large number of correlations, we applied both the Bonferroni [50] and Benjamini–Hochberg corrections [51], the latter of which was included as an alternative to the highly conservative Bonferroni correction. Three correlations remained significant after the Benjamini–Hochberg correction. As the number of stars collected during the active trials increased, so did memory accuracy (*r* = 0.39, *p* = 0.009); this relationship held only for active but not passive encoding trials. During encoding, there were occasions where the participant stopped along the route (during both active and passive trial types); we measured the distance of a stop from the nearest intersection, and found that as this distance got smaller, memory accuracy increased (*r* = −0.39, *p* = 0.01). Similarly, we measured this distance during the retrieval phase (when a participant was re-tracing their route), and found the same relationship (*r* = −0.38, *p* = 0.01). The relationships the distances of the stops from the intersections and Route Overlap held only for the active, and not the passive encoding trials. 

Regression analyses. Step-wise regression analyses were used to examine whether any of the factors considered in the correlational analyses (see Table 1) significantly predicted participants’ Route Overlap accuracies following active or passive encoding. 

In Step 1 of the analysis of performance following active encoding, Age entered into the regression equation, and was significantly related to Route Overlap, *F*(1,42) = 75.86, *p* < 0.001. At Step 2, the Hooper Visual Orientation test performance was entered into the equation, *t* = 3.05, *p* = 0.004, and at Step 3, the distance of participants’ stops from the nearest intersection during encoding was entered, *t* = −2.18, *p* = 0.035. At Step 4, the number of stars collected failed to be entered into the equation, *t* = 1.94, *p* = 0.06. The final model significantly predicted Route Overlap, *F*(3, 40) = 38.00, *p* < 0.001, with an *R*^2^ of 0.740, decreasing with Age (β = −0.56) and distance from the nearest decision point (β = −0.19), and increasing with the Hooper performance (β = 0.27).

In Step 1 of the passive encoding analysis, Age entered into the regression equation and was significantly related to Route Overlap, *F*(1,42) = 101.82, *p* < 0.001. At Step 2, the Hooper Visual Orientation test performance was entered into the equation, *t* = 2.24, *p* = 0.031, and at Step 3, the length of the path travelled at retrieval failed to be entered into the equation, *t* = −1.60, *p* = 0.12. The final model significantly predicted Route Overlap, *F*(2, 41) = 58.26, *p* < 0.001, with an *R*^2^ of 0.74, decreasing with Age (β = −0.68) and increasing with Hooper performance (β = 0.24). 

## 7. Discussion

In this study we created a series of virtual environments in which younger and older adult participants travelled routes and then later re-traced those routes, allowing us to assess spatial memory performance. Critically, participants either traveled routes during encoding by active exploration through the volitional control of their movements, or by passively viewing a guided tour. As expected, we found that actively navigating through virtual environments resulted in more accurate performances in re-tracing the travelled routes, than passively viewing a recorded navigation during encoding. As expected, those with higher scores on the Hooper Visual Organizational test had more accurate spatial memories, regardless of how the routes were encoded. Importantly, we showed that the memory enhancement from active encoding was larger for older than younger adults, indicating that age-related deficits in spatial memory can be reduced by active encoding. Our analysis also revealed that the cognitive approach of stopping close to an intersection benefitted later spatial memory following active encoding. We suggest that active encoding provides a high degree of environmental support for older adults, by prompting cognitive engagement with the route information, which can serve to improve encoding and subsequent retrieval of that information.

The positive relationship found between memory accuracy and scores on the Hooper Visual Orientation test suggests that individuals who can create better mental visualizations are better at creating a mental image of the travelled routes, which benefits performance when retracing those routes. The Hooper Visual Orientation test measures one’s ability to mentally rotate and piece together misarranged visual information to identify the depicted object [45,46]. As such, it may be the case that individuals with higher scores on this test are better at mentally translating and combining the visual components of travelled sections of the route, to create a survey representation of the environment. Indeed, this relationship emerged for both active and passive encoding, which indicates that regardless of how one interacts with an environment, the ability to visualize a mental map supports the memory retrieval of route information. 

The finding that older adults’ spatial memory performance was enhanced following active relative to passive encoding, is consistent with other findings in the literature [15,25,26]. For example, Sauzéon and colleagues [26] demonstrated that older adults had better memories for objects in a virtual apartment following active exploration rather than a passively guided tour. Additionally, active encoding has been found to benefit older adults’ memory for spatial information [15,25] showing that when participants are active ‘drivers’, their memory is better than when they act as passive ‘passengers’ in virtual environments. Our findings build on this previous work, by demonstrating that when attempting to retrace a route that is encountered on only one previous occasion, having actively been in control of the initial navigation benefitted memory more than passive guidance. These findings contribute to the literature, suggesting that age-related deficits in spatial memory abilities can be ameliorated through the use of active encoding strategies for navigation. 

The finding that memory accuracy was related to the number of stars collected following active, but not passive encoding suggests that during active navigation, attention is indeed oriented towards information that is relevant to the traveled route. Notably, no relationship was found between memory accuracy and the length of the path traveled, meaning that the collection of stars must have provided a unique benefit towards encoding. One possibility is that a memory benefit was obtained through intrinsic motivation or curiosity [35,36] associated with collecting the stars. As described in the introduction, it has been suggested that momentary feelings of curiosity boost memory and executive functions [37], because phasic activation of the noradrenergic and dopaminergic systems modulate hippocampal activity [38]. Regardless of the potential mechanism, it is clear from our results that having a larger number of interesting or notable targets in the environment during encoding enhanced memory recall. 

We also found a relationships between mental accuracy and the distance of stops from the nearest intersection during encoding and retrieval, such that stopping near an intersection was related to better performance in retracing the route. Furthermore, in the regression analysis, stopping near an intersection during active encoding was found to be predictive of the accuracy in route overlap. When participants stopped moving near an intersection within the environment during encoding, it facilitated their later memory in recalling the spatial route travelled. It may also be the case that the intersections, or decision points, are critical components of the overall spatial representation of the route. Enhanced encoding of these points likely led to a better spatial representation of the details of the route. When the participants stopped moving in the environment during retrieval, they were possibly attempting to remember the decisions that were previously made at the intersection during encoding, and correcting their planned route if necessary. However, it is important to note that only stopping near an intersection during active encoding, and not retrieval, was a significant predictor of route overlap accuracy. This finding suggests that stopping to focus attention on decision points during exploration in the environment aids spatial memory performance. 

In older adults, links have been found between poor spatial representation abilities and declines in both executive functioning [52,53] and episodic memory [27]. The effortfulness hypothesis [54], which states that, because of age-related sensory limitations, there are more cognitive resources that are allocated to the identification of perceptual information, rather than to the encoding of meaningful content, could explain why spatial memory deficits occur in older adults. For example, if limited cognitive resources are directed towards the identification or perceptual processing of a landmark, encoding of the spatial location of the landmark or of the route travelled would be hindered. Indeed, navigational strategies are effortful and cognitively demanding, requiring executive functioning, spatial abilities, and memory [43], which are well-documented as being in decline in older adults. Critically, however, it should be possible to reduce age-related differences in spatial memory performance by implementing encoding strategies that direct cognitive resources specifically to the encoding of spatial information. While our task involved physical components (motor control for locomotion, proprioceptive, and vestibular sensory information) from pressing the movement button and spinning the chair to change directions, it was likely not difficult or demanding enough to create a cognitive burden on the older adults. It would be interesting for future work, to include a divided attention condition, in which participants need to respond to a secondary task while simultaneously navigating during encoding, to determine if heavily taxing their available cognitive resources results in a diminished effect of active encoding. 

Notably, we found that older adults had significantly poorer spatial visualization abilities than younger adults, which may be linked to a common deficit that is associated with the poorer spatial memory performance observed in this age group. Older adults have been found to rely more on egocentric rather than allocentric representations during navigation [55,56], the latter of which involves a more highly developed survey representation of space [57], and this is possibly supported by processes that are similar to those supporting general spatial visualization functions. Evidence suggests that degradation in the hippocampal region is related to declines in survey abilities in older adults [58,59] and also to visual perceptual processing [60], which may be involved in spatial scene construction abilities that are supported by the hippocampus [61,62]. Indeed, older adults have been shown to have worse performances than younger adults in both spatial and scene construction tasks that involve imagining an event [63]. An interesting avenue for future work would be to investigate the links between visual imagery abilities, scene construction, and spatial representations, and how these change in the aging brain.

## 8. Conclusions

The results from this study demonstrate that actively navigating, rather than passively encoding environments results in a more accurate performance when re-tracing a travelled route. Importantly, the magnitude of the memory enhancement from active relative to passive encoding was larger for older than younger adults, reducing the commonly observed age-related deficit in spatial memory performance. Additionally, our findings demonstrate that higher scores on the Hooper Visual Organizational test, and pausing near intersections during active encoding are related to, and predictive of, route overlap accuracy, illuminating the cognitive components that contribute to the benefits of active navigation memory. Overall, our findings suggest that older adults can improve their spatial memory performance for travelled routes by actively controlling their exploration of new environments. 

## Figures and Tables

**Table 1:** Correlations between memory accuracy (percent overlap values) following active and passive encoding, and various neuropsychological factors, as well as task performance measures.

	Active	Passive
SBSD	*r* = −0.07, *p* = 0.62	*r* = 0.11, *p* = 0.48
Hooper	*r* = 0.51, *p* = 0.001 ^a,b^	*r* = 0.42, *p* = 0.005 ^b^
Number of stars passed during encoding	*r* = 0.39, *p* = 0.009 ^b^	*r* = 0.09, *p* = 0.57
Number of intersections passed during encoding	*r* = −0.006, *p* = 0.97	*r* = 0.10, *p* = 0.52
Length of the path travelled at encoding	*r* = −0.09, *p* = 0.57	*r* = 0.03, *p* = 0.83
Length of the path travelled at retrieval	*r* = −0.004, *p* = 0.98	*r* = −0.13, *p* = 0.41
Total duration of stopped movement during encoding	*r* = 0.06, *p* = 0.72	*r* = 0.03, *p* = 0.86
Number of stops during encoding	*r* = 0.14, *p* = 0.36	*r* = −0.03, *p* = 0.85
Distance of stops from the nearest intersection during encoding	*r* = −0.39, *p* = 0.01 ^b^	*r* = −0.01, *p* = 0.96
Total duration of stopped movement during retrieval	*r* = −0.23, *p* = 0.13	*r* = 0.06, *p* = 0.70
Number of stops during retrieval	*r* = 0.04, *p* = 0.82	*r* = 0.01, *p* = 0.95
Distance of stops from the nearest intersection during retrieval	*r* = −0.38, *p* = 0.01 ^b^	*r* = −0.25, *p* = 0.11

Both the Bonferroni and Benjamini–Hochberg corrections for multiple comparisons were conducted; SBSD = Santa Barbara Sense of Direction scale; ^a^ Correlations were significant following Bonferroni correction; ^b^ Correlations were significant following Benjamini–Hochberg correction.
